# Sand Particle Size and Phosphorus Amount Affect *Rhizophagus irregularis* Spore Production Using In Vitro Propagated Spore as a Starter Inoculum in Rhizosphere of Maize (*Zea mays*) Plantlets

**DOI:** 10.3390/jof7100846

**Published:** 2021-10-09

**Authors:** Pulak Maitra, Jubair Al-Rashid, Nirmal Chandra Barman, Md. Niuz Morshed Khan, Dipa Mandal, Noorain Munim Rasul, Apu Chowdhury, Ahmed H. El-Sappah, Jia Li

**Affiliations:** 1 Solid-State Fermentation Resource Utilization Key Laboratory, Faculty of Agriculture, Forestry and Food Engineering, Yibin University, Yibin 644000, China; pulak@yibinu.edu.cn (P.M.); apuchowdhury@yibinu.edu.cn (A.C.); Ahmed_elsappah2006@yahoo.com (A.H.E.-S.); 2 Apex Biotechnology Laboratory, Apex Holdings Ltd., Gazipur 1751, Bangladesh; nirmal.bge@gmail.com (N.C.B.); niuzkubge12@gmail.com (M.N.M.K.); noorain@apexbiofertilizer.com (N.M.R.); 3 Institute of Microbiology, University of Chinese Academy of Sciences, Beijing 100101, China; dipaiuanft112@mails.ucas.edu.cn

**Keywords:** arbuscular mycorrhiza, *R. irregularis*, monxenic culture, phosphorus treatment, inoculum production

## Abstract

Microbial inoculants, particularly arbuscular mycorrhizal (AM) fungi, have great potential for sustainable crop management. In this study, monoxenic culture of indigenous *R. irregularis* was developed and used as a tool to determine the minimum phosphorus (P) level for maximum spore production under the in vitro conditions. This type of starter AM fungal inoculum was then applied to an in vivo substrate-based mass-cultivation system. Spore production, colonization rate, and plant growth were examined in maize (*Zea mays* L.) plant inoculated with the monoxenic culture of *R. irregularis* in sand graded by particle size with varying P levels in nutrient treatments. In the in vitro culture, the growth medium supplemented with 20 µM P generated the maximum number of spores (400 spores/mL media) of *R. irregularis*. In the in vivo system, the highest sporulation (≈500 spores g^−1^ sand) occurred when we added a half-strength Hoagland solution (20 µM P) in the sand with particle size between 500 µm and 710 µm and omitted P after seven weeks. However, the highest colonization occurred when we added a half-strength Hoagland solution in the sand with particle sizes between 710 µm and 1000 µm and omitted P after seven weeks. This study suggests that substrate particle size and P reduction and regulation might have a strong influence on the maximization of sporulation and colonization of *R. irregularis* in sand substrate-based culture.

## 1. Introduction

Arbuscular mycorrhizal (AM) fungi form a mutualistic symbiosis with roots of more than 80% of the terrestrial plant species [1]. Plants can access nutrients and water from a much larger volume of soil using AM fungal mycelia than only their roots would allow [2]. AM fungi can help plants acquire phosphorous (P), nitrogen (N), potassium (K), zinc (Zn), and copper (Cu) [3,4,5,6,7,8]. They also improve plant drought tolerance by improving water transportation, osmotic adjustment, gas exchange, and protection against oxidative damage [9,10,11]. AM fungi form soil aggregates and improve soil structure [12]. Therefore, AM fungi have great potential for sustainable agriculture, and their applications in agriculture have increased in recent years [13,14,15]. 

The in vitro culture of mycorrhizal mono species is critically important for pure inoculum production. These cultures can be used for subsequent bulk AM fungal inoculum production for agricultural use. However, to date, only a few AM fungal species have been successfully grown using the root organ culture. *Agrobacterium rhizogenes* induced transformed roots of *Daucus carota* L. increases spore production of *Funneliformis mosseae* and *R. irregularis* in vitro [6]. Among several AM fungal species, *R. irregularis* appears to be very promising based on its cultural characteristics, sporulation ability, and in terms of plant growth and yield potential [16]. However, the obligate bio-trophic nature of AM fungi has complicated the development of cost-efficient large-scale production methods to achieve high-quality inoculum of AM fungi [17]. Therefore, large-scale production of high-quality pure AM fungal inoculum using easily available materials is essential for its wide-scale application.

In previous studies, several methods were demonstrated to be effective for large-scale inoculum production of AM fungi. These include monoxenic or in vitro cultures [18,19,20], conventional pot cultures in the greenhouse [21], the aeroponic system, and nutrient film techniques [22]. The major limitation of these methods is the low number of spores generated and the presence of contaminants or mixed species inoculum. Furthermore, the long-time storage and maintenance of propagules in the synthetic medium demand expert hands that are expensive. Previous studies have demonstrated that substrate-based methods such as pot cultures resulted in the easy production of a large amount of AM fungal inoculum having higher colonizing efficiency in the host plants [23]. Sand or vermiculite can be used as a substrate for AM fungal spore production [24]. Particle size is a crucial factor in the substrate-based multiplication of AM fungi. Optimum soil aeration, which mainly depends on substrate particle size, is essential for the establishment and metabolic activity of AM fungi [25]. Substrate nutrient levels should support the survival of the host plants but need to be low enough to allow the fungus to infect plant roots, and form spores abundantly [26,27]. Nutrient solutions without or with low levels of P have often been reported as beneficial for AM fungal root colonization and spore production [8,28]. Numerous studies have been conducted on the optimization of spore production of AM fungi in vivo [5,8,23,24]. Nevertheless, in vivo AM fungal spore production using aseptic monoxenic culture as a starter inoculum has not been investigated. Furthermore, the effect of substrate (sand) particle size and P amount on maximization of sporulation of AM fungi was not investigated in sand substrate-based culture. 

In this study, we optimized several physical and chemical parameters for high-quality AM fungal inoculum production using maize as a host plant in the sand. Monoxenic culture of the AM fungal species was established from the spores isolated from a maize field soil of Gazipur, Bangladesh. These were used to inoculate the maize plants. River sand was graded into different ranges of particle sizes and used as the substrate. We also verified the benefits of reducing phosphorus content of the nutrient solution and that of phosphorus omission after 7-weeks of growth of the plants. The aim of this study was as follows: (i) establishing a monoxenic culture of indigenous AM fungal species; (ii) testing the effect of substrate (sand) particle size on maximization of AM fungal spore production and colonization on maize (*Zea mays* L.) plant system; (iii) testing the effect of nutrient strength and P shortage on AM fungal sporulation. We hypothesized that sand particle size, nutrient strength, and P absence might influence AM fungal sporulation and colonization using maize as a host plant in the sand substrate-based culture. 

## 2. Materials and Methods

### 2.1. Isolation of AM Fungal Spore

Soil samples were collected from maize fields from Gazipur, Bangladesh (24°06′10.9″ N, 90°16′19.5″ E). We weighed 100 g soil in a 1 L plastic beaker with 200 mL of water. Soil samples were wet-sieved through a cascade of sieves with 300, 106, and 45 µm cutoffs placed on a plastic bowl. The soil collected on 106 µm and 45 µm sieves was placed in a 50 mL tube. The tube containing soil and water from the 45 µm sieve was centrifuged for 5 min at 3000 rpm. The supernatant along with floating debris was discarded. Then, 45% sucrose was added to the tube, the tube was shaken well and centrifuged for 2 min at 1800 rpm. The sucrose supernatant containing spores was poured on sieves with cutoffs 106 µm and 45 μm in a bowl. Material collected between 106 µm and 45 µm was collected to isolate AM fungal spores and poured on a Petri dish. Spores were picked up under a dissecting microscope using a micropipette. Healthy hyaline spores were selected (Supplementary Figure S1). The collected spores were placed in a microfuge tube in Ringer’s solution and stored at 4 °C.

### 2.2. Establishment of Monoxenic Culture of AM Fungi

Wild type *A. rhizogenes* is a Gram-negative soil bacterium containing plasmids that can induce the formation of transformed roots able to grow in hormone-free media. It stimulates the formation of proliferative, multi-branched, adventitious roots at the infection site of dicotyledonous plants [29]. We obtained *A. rhizogenes* ICMP-8640 from ICMP culture collection, Landcare Research-Manaaki Whenua, New Zealand. Fresh carrots were surface-sterilized, cut into discs, and co-cultivated with *A. rhizogenes* ICMP-8640. Carrot (*Daucus carota* L.) discs were transferred to ½ MS agar medium [30] containing 500 mg L^−1^ cefotaxime for two days. All discs formed roots after 10–15 days. Root tips (2–3 cm) were transferred to MSR agar with 150 mg L^−1^ cefotaxime and were sub-cultured into the same media after 12–18 days, to remove *A. rhizogenes*. Contamination-free healthy roots were obtained after 3–5 successive subcultures. Vigorous geo-negative roots with fishbone structure were detected after 20–25 days of cultivation on MW (Modified White’s Medium) [31]. Root clone C-8 was selected from numerous root clones because of its proliferative growth in media (Supplementary Figure S2A). The composition of MW medium is summarized in Supplementary Table S1.

We surface-sterilized AM fungal spores in a 1.5 mL tube in a laminar airflow hood. The tube containing the AM fungal spores was centrifuged briefly (3–5 s), the Ringer’s solution was discarded using a micropipette, and the spores were rinsed twice with sterile water. The spores were first surface sterilized with 2% Chloramine-T (with 2–3 drops of Tween-20 per 100 mL solution) for 10 min. The spores were centrifuged briefly and rinsed three times with sterile water. The spores were then disinfected with an antibiotic solution (0.02% Streptomycin sulfate and 0.01% Gentamycin sulfate, filter sterilized) for 10 min. Intact spores were selected under a dissection microscope inside a laminar airflow hood and placed on 0.7% water agar for germination. Germinated single spores (Supplementary Figure S2B) were placed near C-8 roots in minimal (M) media [32] containing 0.4% Phytagel (Sigma, St Louis, MO, USA). The Petri dishes (diameter: 9 cm) were incubated in inverted conditions at 27 °C ± 2 in the dark. After five weeks, they were checked under a compound microscope (Axio Imager A1, Carl Zeiss, Germany) at 80X magnification for the establishment of monoxenic culture, and spore formation. We found that hyphae had spread all over the plate (Supplementary Figure S2C) and secondary spores had formed (Supplementary Figure S2D). We successfully obtained three monoxenic cultured isolate of AM fungi. Fresh cultures were prepared using segments of the initial monoxenic culture as inoculum and checked for root growth and spore formation after five weeks. Monoxenic cultures were considered to be stable in continuous culture after several cycles. Every 7 weeks, several colonized carrot root segments (2–3 cm long) were transferred to a fresh Petri dish (diameter: 9 cm) containing M medium. This regular sub-culturing ensured satisfactory growth of the AM-colonized roots. The composition of M media is given in the Supplementary Table S2.

### 2.3. Molecular Identification of Monoxenic Mycorrhizae

Three successfully established monoxenic cultured isolate of AM fungi were identified by sequencing of the D2 LSU gene. Briefly, the genomic DNA was isolated from the spores of monoxenic cultures of AM fungal isolate Apex-MYK-01, Apex-MYK-02, and Apex-MYK-Gd-01 using PrepMan^®^Ultra sample preparation reagent kit (Applied Biosystems, Foster City, CA, USA). PCR Master Mix of fast MicroSeq^®^ D2 LSU rDNA fungal PCR kit (Applied Biosystems, Foster City, CA, USA) was used and the products were purified by E.Z.N.A Gel Extraction Kit (Omega Bio-Tek, Norcross, GA, USA). Cycle sequencing was carried out with the purified PCR product and the forward primer reaction Mix of MicroSeq^®^ D2 LSU rDNA fungal sequencing kit (Applied Biosystems, California, USA). The cycle sequencing products were purified by E.Z.N.A Gel Extraction Kit (Omega Bio-Tek, Norcross, GA, USA), and dissolved in Hi-Di™ formamide (Applied Biosystems, Foster City, CA USA). They were denatured and sent for sequencing to the National Institute of biotechnology (NIB), Savar, Dhaka. The sequences were analyzed using 3130 Genetic Analyzer 4-capillary sequencer using big dye 3.1 chemistry. DNA similarity was analyzed using NCBI BLAST server (http://www.ncbi.nlm.nih.gov, accessed on 10 June 2021). The monoxenic cultures Apex-MYK-01, Apex-MYK-02, and Apex-MYK-Gd-01 were identified as *R. irregularis* (MZ424786), *Rhizophagus fasciculatus* (MZ424827), *R. fasciculatus* (MZ433185), respectively. The sequences of these three isolates were submitted to the GenBank database and the corresponding accession numbers are indicated in parenthesis. Then, we selected *R. irregularis* isolate (Apex-MYK-01) for further experiments.

### 2.4. Optimization of M-Media for R. irregularis Isolate Apex-MYK-01 

Monoxenic cultures of *R. irregularis* isolate Apex-MYK-01 were selected based on vigorous root growth and spore formation. Segments (size 2 cm^2^) containing C-8 roots with fishbone structure and numerous spores were cut out of the *R. irregularis* monoxenic cultures and placed on a sterile Petri dish. M media with five different phosphorus concentrations, i.e., 40, 35, 30, 20, and 10 µM were prepared with 0.4% Phytagel (Sigma, St Louis, MO, USA). In each Petri dish, 20 mL of medium was poured and prepare 10 replicate of M-medium each with different P concentration. The Petri dishes were incubated in inverted conditions at 27 ± 2 °C in the dark. After 7 weeks, *R. irregularis* monoxenic cultures were dissolved in 10 mM sodium citrate buffer and sieved through a 38 μm sieve. The dissolved spore were then counted according to Maitra et al. [33].

### 2.5. Sand Substrate-Based Inoculum Production of R. irregularis Isolate Apex-MYK-01 under Maize Plant System 

#### 2.5.1. Sand Processing and Grading

A fine aggregate sand sample was collected from Sunamganj, Bangladesh. The sand has low absorbance, the color is mainly reddish, the percentage of coarse particles is comparatively high, and it is free from clay and organic matters. The sand sample was sieved through mesh no BSS 12 (1.40 mm) by manual sieving for 1 min. Each 10 kg of sieved sand was washed in running tap water for about 20 min to remove any debris. The bulk sand sample was surface cleaned with a freshly prepared 10% solution of commercial bleach (5.25% sodium hypochlorite) for about 1 h with a few drops of liquid detergent Tween 20 added to the mixing tank. Then, the sand was thoroughly rinsed 4–5 times with deionized water. The sand was acid washed using 0.1M HCl solution to remove organic matter and nutrients. The sand was incubated with the acid solution at room temperature for 24 h, then thoroughly rinsed 4-5 times with distilled water to reduce the concentration of HCl to less than 5 mM, and then oven-dried for 48 h at a constant temperature (70 °C ± 2).

Processed sand was graded into four texture grades using a nest of sieves (British standard size, BSS (410/1969) Mesh No): 16 (1000 µm), 22 (710 µm), 30 (500 µm), 52 (300 µm) and 150 (106 µm). The following particle size classes were produced: grade A (particles that pass through 16 BSS sieve and are retained on 22 BSS sieve) >710 µm~<1000 µm; grade B (particles that pass through 22 BSS sieve and are retained on 30 BSS sieve) >500 µm~<710 µm; grade C (particles that pass through 30 BSS sieve and are retained on 52 BSS sieve) >300 µm~<500 µm; grade D (particles that pass through 30 BSS sieve and are retained on 52 BSS sieve) >106 µm~<300 µm. Graded sand (Supplementary Figure S3) was autoclaved (121 °C for 2 h at 15 PSI) for two consecutive days and stored for further use. The description of physical properties and water holding capacity of different graded sand is summarized in Supplementary Table S3.

#### 2.5.2. Inoculation of Maize Seedlings in Sand with *R. irregularis* Isolate Apex-MYK-01

The spores of the established monoxenic culture of AM fungus (*R. irregularis* isolate Apex-MYKE-01), was used as primary inoculum for large-scale AM fungal inoculum production in sand-substrate based culture for agricultural use. Maize plants grown in the sand were used as host. Healthy maize (BARI hybrid maize-9 released by Bangladesh Agriculture Research Institute in 2007) seeds were surface sterilized and germinated on moist filter papers. Seedlings were transplanted at 2 cm depth in sterilized sand in pots when they were about 2 days old. Gel plugs containing 30 ± 5 spores were cut out from monoxenic cultures of the AM fungal strain and added to the rhizosphere zone of the maize seedlings. All maize plants were grown in plastic pots (17 cm in diameter and 18 cm height) in a greenhouse and maintained at 28 ± 2 °C temperature with 68 ± 5% relative humidity. Modified Hoagland’s nutrient solution (20 mL/kg sand) was added to the pots every day for the growth of the maize plants. Hoagland’s nutrient solution [34] of different strengths (i.e., full-strength Hoagland’s solution with 40 μM P and half-strength Hoagland’s solution with 20 μM P) was used to optimize maximum spore formation. The concentration and composition of full-strength and half-strength Hoagland’s nutrients solution are described in Supplementary Table S4. Four treatments were designed in which Hoagland’s nutrient solutions of different strength were used, and P was either omitted after 7 weeks of growth on the maize plants or not. These include full-strength Hoagland (P), full strength Hoagland with P omission (−P), half-strength Hoagland (P), and half-strength Hoagland with P omission (−P) nutrient treatments. The sand of 4 texture grade was used. In total, 96 pots (4 nutrient treatments × Sand 4 texture grades × six replicates) were included in this study. The detailed information about nutrient application is summarized in Supplementary Table S5. 

### 2.6. Harvest and Data Measurement 

Plants were harvested after 13 weeks. Shoot weight and root weight were measured to determine the vegetative growth of the maize plants in different treatments. Spore density and colonization were measured according to Maitra et al. [33,35]. Briefly, we suspended 20 g air-dried sand using 250 mL distilled water in a flask and sieved through 1 mm and 38 µm sieves. Soil with spores on 38 µm sieve was transferred into a 50-mL tube with 15 mL distilled water and centrifuged for 3 min at 700 g. After supernatant was discarded, pellet was suspended with 15 mL sucrose solution (500 g L^−1^) and centrifuged for 3 min at 250 g. The supernatant was filtered through a 38 μm sieve, and spores on the sieve were washed into a Petri dish plate and counted under ×40 magnification (Nikon 80i, Tokyo, Japan).

Root segments (c. 1 cm long) were boiled in 10% KOH solution at 92 °C for 25 min, neutralized with 2% HCl solution at room temperature for 5 min, and stained with 2% trypan blue-lactoglycerol solution dye at 92 °C for 2 min to measure the percentage of root length colonized by AMF. Forty-five root segments were randomly selected from each sample and examined for the percentage of root length colonized by AM fungi using the magnified intersection method with 450 fields of view per sample at ×200 magnification [36].

### 2.7. Statistical Analysis

All statistical analyses were conducted in R2.15.1 [36]. Tukey’s honestly significant difference (HSD) test was used to examine the significant difference of the spore number in monoxenic plates of *R. irregularis* in M medium containing 40, 35, 30, 20 and 10 µM P. Three way ANOVA or Kruskal–Wallis tests were used to explore the influence of sand particle size, nutrient strength and phosphorus omission on the spore number and colonization rate of *R. irregularis* and shoot and root weight of maize plant based on data normality and homogeneity. Significant differences among different treatments were further tested using Tukey’s HSD test at *p* < 0.05 level. For the data that did not satisfy the normality of distribution or homogeneity of variance after transformation, a nonparametric Kruskal–Wallis test was used to examine the effect of fertilization and liquid inoculant, followed by Conover’s test using the ‘post hoc.kruskal.conover.test’ function in the PMCMR package [37].

## 3. Results

### 3.1. Monoxenic Culture and Identification of AM Fungal Strain

The monoxenic culture of AM fungal strain with the typical structure of hyphal branching and spore formation was successfully established in M medium with Ri T-DNA-transformed carrot roots (Supplementary Figure S2D). Nucleotide BLAST results demonstrated that the query sequence of the established monoxenic AM fungal isolate Apex-MYK-01 (MZ424786) acquired in the current study is 99% identical to the representative sequences of *R. irregularis* in the GenBank (FR750090, FR750130). 

### 3.2. AM Fungal Spore Number in Different Level of P in Monoxenic Culture 

**Figure 1:**
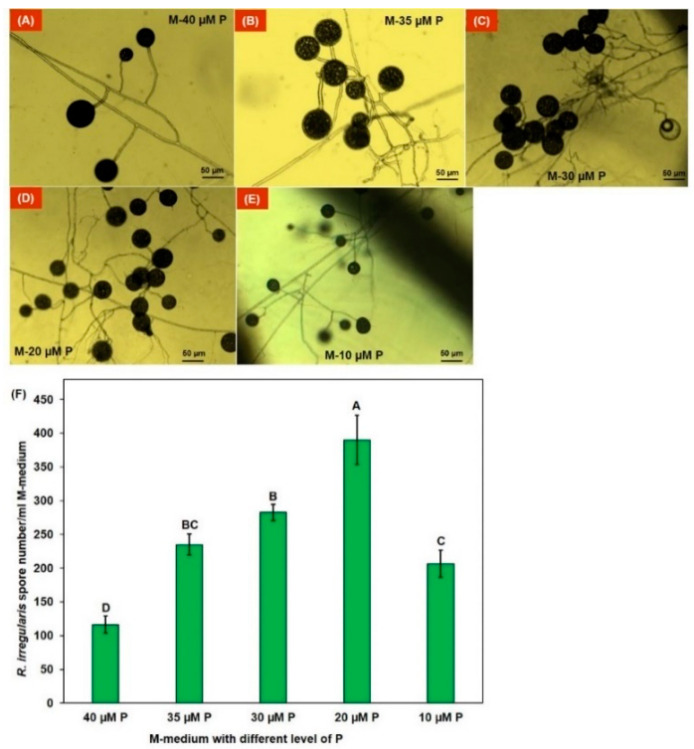
Monoxenic culture of *R. irregularis isolate* Apex-MYK-01 in M- medium with different phosphorus (P) concentration in the medium (**A**–**E**) and sporulation rate (**F**).

The AM sporulation was influenced by the P concentration in the M medium. For instance, higher spore formation was observed in monoxenic culture of *R. irregularies* in M medium with 20 µM P (Figure 1). We found that the *R. irregularis* spores in 40 μM P M media were the largest in size but fewer in number, and those in the 10 μM P M media were the smallest (Figure 1; Supplementary Table S6; Supplementary Figure S4). Roots in the 40 μM P plates were thick but were turning brown, and those in the 10 μM P plates were thin. The plates containing M media with phosphorus concentrations between 20 and 35 µM had fresh white roots with a higher number of *R. irregularis* spores. The highest number of spores per ml was obtained in monoxenic cultures with 20 μM P (Figure 1).

### 3.3. R. irregularis Spore Density and Colonization Rate in Sand Substrate-Based Culture

**Figure 2:**
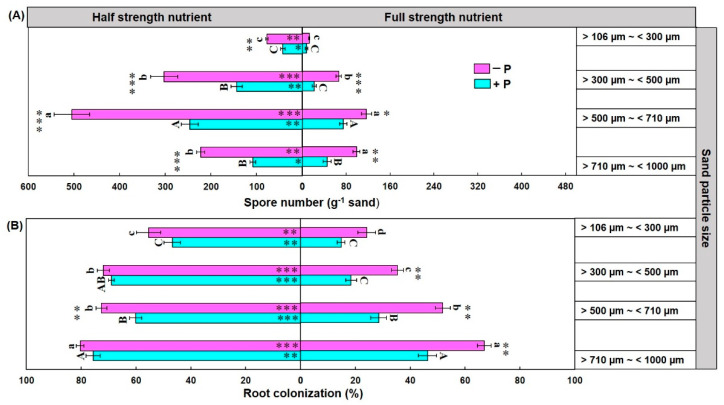
Effect of sand particle size, nutrient strength and phosphorus omission on spore number (**A**) and colonization rate (**B**) of *R. irregularis* grown with maize plant in sand. Data are means ± SE (*n* = 6). Bars without shared uppercase and lowercase letters indicate significant difference of spore number and root colonization rate among different particle size of sand according to Tukey’s HSD test at *p* < 0.05. Bars with asterisks represent significant difference of spore density and colonization between phosphorus omission (−P) and phosphorus without omission (+P) treatments. Asterisks within column indicate significant difference of spore number and colonization rate between half strength and full strength nutrient treatments. * *p* < 0.05, ** *p* < 0.01, *** *p* < 0.001.

The Kruskal–Wallis test showed that sand particle size, Hoagland’s nutrients strength, and phosphorus omission significantly influenced the spore formation of *R. irregularis* in maize plant sand substrate-based culture (Table 1). The spore number was significantly higher in the sand with particle size >500 µm~< 710 µm compared to the other particle sizes of sand in half-strength nutrient treatment (Figure 2A). However, the highest number of spores (≈500) was observed in phosphorus omission treatment under half-strength nutrient treatment in the sand with particle size >500 µm~<710 µm (Figure 2A). Furthermore, a significantly higher spore number was observed in half-strength nutrient treatment compared to the full-strength nutrient treatment in all sand under different phosphorus treatment (Figure 2A). Moreover, a significantly higher spore number was observed in phosphorus omission treatment compared to the treatments in which P was supplied in the sand of different particle sizes (Figure 2A).

**Figure 3:**
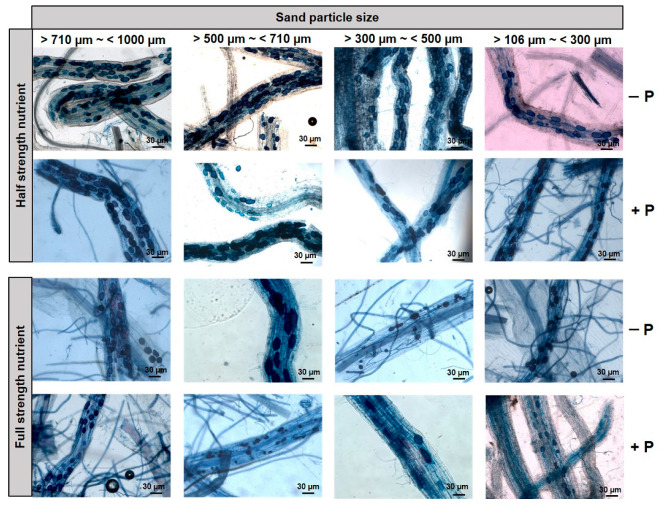
Root colonization of *R. irregularis* under different treatments with different sand particle used to grow maize.

The Kruskal–Wallis test showed that sand particle size, Hoagland’s nutrients strength, and phosphorus omission significantly influenced the colonization rate of maize plant inoculated with *R irregularis* in the sand substrate-based culture. The colonization rate was significantly different in different particle sizes of sand of maize plant root and the highest colonization was observed in the sand with particle size >710 µm~<1000 µm (Figure 2B and Figure 3). Additionally, root colonization was significantly higher in half-strength nutrient treatment compared to the full-strength nutrient treatment in the sand of all particle sizes both in P omission and P without omission treatments (Figure 2B and Figure 3). Moreover, higher colonization was observed in P omission treatment compared to the treatments without P omission in the sand of different particle sizes >710 µm~<1000 µm, >500 µm~<710 µm and >300 µm~<500 µm (Figure 2B and Figure 3).

### 3.4. Maize Shoot and Root Weight and Their Relation with Spore Density and Colonization

**Figure 4:**
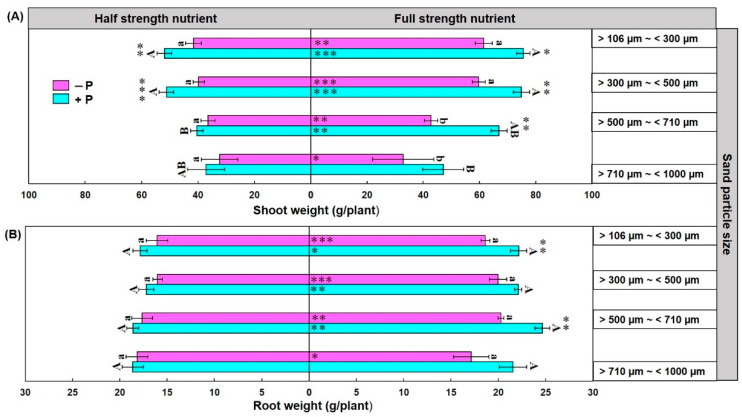
Effect of sand particle size, nutrient strength and phosphorus omission on shoot weight (**A**) and root weight (**B**) of maize plant grown with *R. irregularis* inoculum in sand substrate-based culture. Data are means ± SE (*n* = 6). Bars without shared uppercase and lowercase letters indicate significant difference of shoot weight and root weight among different particle size of sand according to Tukey’s HSD test at *p* < 0.05. Bars with asterisks represent significant difference of shoot weight and root weight between phosphorus omission (−P) and phosphorus not omission (+P) treatments. Asterisks within column indicate significant difference of shoot weight and root weight between half strength and full strength nutrient treatments. * *p* < 0.05, ** *p* < 0.01, *** *p* < 0.001.

Three-way ANOVA showed that sand particle size, Hoagland’s nutrients strength, and phosphorus omission significantly influenced the shoot weight of maize plant sand substrate-based culture (Table 1). The higher shoot weight was observed in maize plants grown in sand particle size >100 µm~<300 µm and >300 µm~<500 µm compared to sand particle size >500 µm~<700 µm and >700 µm~<1000 µm. Furthermore, significantly higher shoot weight was observed in full-strength nutrient treatment compared to the half- strength nutrient treatment. However, significantly higher shoot weight was observed in P without omission treatment compared to the P omission treatment in maize plant grown under sand particle size >100 µm~<300 µm and >300 µm~<500 µm (Figure 4A).

**Figure 5:**
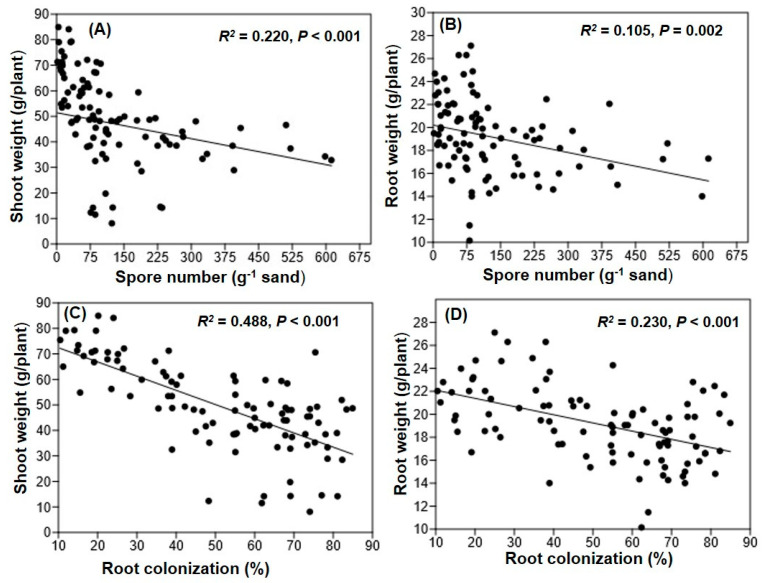
Linear relationships of shoot and root weight with spore number (**A**,**B**) and shoot and root weight with root colonization (**C**,**D**) of *R. irregularis* isolate Apex-MYK-01 inoculaed with maize seedlings grown in sand substrate-based culture.

Three-way ANOVA showed that Hoagland’s nutrients strength and phosphorus omission significantly influenced the root weight of maize plant sand substrate-based culture but sand particle size does not have any effect on root weight (Table 1). Significantly higher root weight was observed in full-strength nutrient treatment compared to the half-strength nutrient treatment (Figure 4B). Furthermore, shoot and root weight of maize plant were significantly correlated with *R. irregularis* spore density and colonization rate (Figure 5).

## 4. Discussion

We initially collected *Glomus sp*. spores from maize field soil rich in AM fungal spores. Monoxenic cultures were developed from these spores, and one isolate was identified as *R. irregularis*. Results show that phosphorus level determines the spore density in the in vitro system. Significantly, 66% higher spore yield was obtained in monoxenic culture when 20 µM KH_2_PO_4_ was used compared to 35 µM KH_2_PO_4_ (control) and followed by 30 µM KH_2_PO_4_. Lower spore yield obtains in both 10 µM and 40 µM KH_2_PO_4_ when applied. These results may be due to the host-dependent mycorrhizal interaction [38]. Reduction of phosphorus in 10µM P treatment lead to minimum root growth compared to control; as a result, spore production was low. Similarly, increased phosphorus level in 40 µM P treatment reduces 50% spore production but, in this case, larger spore size and vigorous root growth were observed.

Maize plants were inoculated with gel plugs containing spores from the monoxenic cultures and grown in the sand of different particle sizes. The result showed that reduction or omission of P from Hoagland solution enhanced both spore production and root colonization rate, regardless of the sand particle size. We found the highest number (≈500) of *R. irregularis* spores when we used half-strength Hoagland solution at regular intervals, and the treatment without P after seven weeks. This may be because low P levels help higher spore production with maximum root colonization percentage [39] as high soil P decreased AM fungal abundance [35]. In each sand particle size grade, spore production and root colonization rate decreased when full-strength Hoagland with 40 µM P was used. Similarly, Millner et al. [40] reported that using modified Hoagland’s solution in maize plants infected with *Claroideoglomus etunicatum*, *F. mosseae*, or *Gigaspora margarita* in a sand substrate-based system resulted in good spore production. Their findings directly support the application of half-strength Hoagland in this experiment. In addition, the timing of nutrient application and omission influenced spore production and colonization levels, as nutrient requirements of cultured AM fungi with maize plants differ throughout its life cycle. Similarly in a previous study, N and P addition influenced the spore production and colonization at different stages of the life cycle of maize plants [41,42]. In our study, P omission at the maize reproductive stage, approximately seven weeks from the transplantation of the seedlings, enhanced spore production with a higher infection percentage.

**Figure 6:**
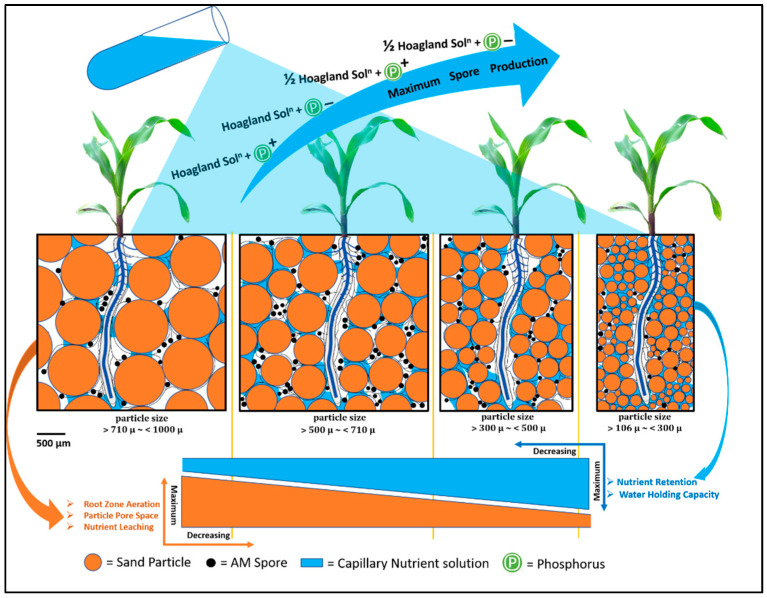
Conceptual framework of in vivo propagation of *R. irregularis* under maize (*Zea mays* L.) plant system in sand substrate-based culture.

Among sand particles, *R. irregularis* spore production was highest in grade B (sand particle size >500 µm~<710 µm) followed by grade C (sand particle size >300 µm~<500 µm) when we used half-strength Hoagland. Gaur et al. [28] demonstrated that the production of infectious propagules was 40% higher for plants grown in a sand-based substrate. However, when we used full-strength Hoagland nutrient, spore production was highest in grade B followed by grade A (sand particles size >710 µm~<1000 µm). Nutrient leaching was lesser in grade C compared to grade A when we used a half-strength Hoagland solution. Maize plants grown in grade-A experienced drought conditions due to their lower water retention capacity as described in Figure 6. When we used full-strength Hoagland, phosphorus leached out of grade-A substrate more easily than from grade C substrate. The high P level may negatively regulated the spore production in grade C. Physical parameters such as adequate aeration, humidity, drainage, and oxygen supply were probably higher in grade A than grade C. These conditions resulted in better spore production. The result showed that spore production was lower when we used the smallest particle size (sand grade D, sand particles size >106 µm~<300 µm). Grade D also had a higher water retention capacity that leads to more frequent nutrient saturation conditions. This nutrient saturation condition may be led to a decrease in AM fungal colonization and spore production as high P concentration decreased *R. irregularis* sporulation in monoxenic culture in our study. Sand grade B had optimal water retention capacity and adequate aeration. These conditions may result in the highest AM fungal spore production among all the nutrition treatments.

Both sporulation and percent root colonization were significantly correlated with root and shoot biomass. This may be because low P availability increased AM fungal colonization rate and plants supply C for growth and maintenance of AM fungi. Furthermore, high P availability down-regulates the expression of several P transporter genes which suppress AM fungal symbiosis [43]. When sufficient P is available, the plant does not need to share carbon for the maintenance of AM fungi for nutrient acquisition. These conditions lead to minimum colonization and higher plant biomass yield [39]. In our study, phosphorus was omitted from the Hoagland solution to stress the maize plants with low nutrient, as a result plant biomass declined, which in turn increased colonization. Therefore, higher root colonization ensures maximum spore density in sand substrate-based culture. Larger sand particle substrate created artificial plant stress because of minimum water holding capacity. In addition, N:P ratios in a particular substrate, especially low nutrient (Primarily P) supply, and the host physiology may regulate plant infectivity and mass production of spore in sand substrate-based system [1,44,45,46].

## 5. Conclusions

In conclusion, we isolated indigenous AM fungal spores, developed monoxenic culture and identified one of the monoxenic strain as *R. irregularis.* We optimized the condition for mass inoculum production of *R. irregularis* in the sand in maize (*Zea mays* L.) plant system with in vitro propagated spore (monoxenic culture) as a starter inoculum. In our study, sand substrate-based culture, nourished with half-strength Hoagland with P omission at plant reproductive stage and sand particle size >500 µm~<710 µm have been adequate for the cultivation of *R. irregularis* species with *Z. mays* as a host with maximum sporulation. This study suggests that substrate particle size and P reduction and regulation might have a strong influence on the maximization of sporulation and colonization of *R. irregularis* in the sand substrate-based culture.

## Figures and Tables

**Table 1:** Kruskal–Wallis test or three way ANOVA examining the effect of sand particle size, nutrient strength and phosphorus omission on the spore density and root colonization rate of *R. irregularis* and shoot and root weight of maize plant grown in sand substrate-based culture.

Source of Variation	d.f.	Spore Density		Colonization		Shoot Weight		Root Weight	
		*χ2*	*P*	*χ2*	*P*	*F*	*P*	*F*	*P*
Sand particle size (SP)	3	39.10	<0.001	27.52	<0.001	17.16	<0.001	2.440	0.070
Nutrient strength (NS)	1	38.48	<0.001	47.64	<0.001	51.22	<0.001	45.33	<0.001
Phosphorus omission (PO)	1	9.360	0.002	7.753	0.005	28.72	<0.001	23.53	<0.001
SP × NS	3	na	na	na	na	2.196	0.039	2.804	0.045
SP × PO	3	na	na	na	na	0.195	0.899	0.242	0.866
NS × PO	1	na	na	na	na	4.144	0.045	6.591	0.012
SP × NS × PO	3	na	na	na	na	0.721	0.542	0.533	0.661

## Data Availability

Not applicable.

## Supplementary Materials

The following are available online at https://www.mdpi.com/article/10.3390/jof7100846/s1, Figure S1: Arbuscular mycorrhizal (AM) fungal spores isolated from soil, Figure S2: Establishment of monoxenic culture of AM fungi, Figure S3: Illustration of pot experiment of maize plant inoculated with *R. irregularis* in different sand grade of sand, Figure S4: Box-plot described the spore size of *R. irregularis* grown in M-Medium supplemented with different level of P, Table S1: Composition of Modified Whit’s (MW) medium, Table S2: Composition of Minimal (M) medium, Table S3: Sand physical properties and water holding capacity, Table S4: Composition of Hoagland’s nutrients solution, Table S5: Hoagland’s nutrient application, Table S6: Average spore size of *R. irregularis* grown in M-medium supplemented with different level of P.
