# Supplementary material for: Sand Particle Size and Phosphorus Amount Affect Rhizophagus irregularis Spore Production Using In Vitro Propagated Spore as a Starter Inoculum in Rhizosphere of Maize (Zea mays) Plantlets
Source: J Fungi (Basel). 2021 Oct 9;7(10):846. doi: 10.3390/jof7100846 (PMC8541049; doi:10.3390/jof7100846)
Supplement: Supplementary file 1 [file jof-07-00846-s001.zip › jof-1381351-supplementary.pdf]

Supplementary table S1 : Composition of Modified Whit's (MW) medium

Nutrient	Final Concentration (mg per liter media)
$MgSO_4.7H_2O$	731
KNO_3	80
KCl	65
Na_2SO_4	199.7
Ca $(NO_3)_2.4H_2O$	288
Glycine	3
Pyridoxine HCl	0.1
Niacin	0.5
Myoinositol	50
Thiamine	0.1
NaFeEDTA	8
$NaH_2PO_4.2H_2O$	21.5
KI	0.75
$MnCl_2.4H_2O$	6
$ZnSO_4.7H_2O$	4.2455
H_3BO_3	1.5
$CuSO_4.5H_2O$	0.13
$Na_2MoO_4.2H_2O$	0.0024
Sucrose	30000

Supplementary table S2: Composition of Minimal (M) medium

Nutrient	Final Concentration (mM per liter media)				
	M media (standard)	M media (10µM P)	M media (20µM P)	M media (30µM P)	M media (40µM P)
$MgSO_4.7H_2O$	3	3	3	3	3
KNO_3	0.79	0.79	0.79	0.79	0.79
KCl	0.87	0.87	0.87	0.87	0.87
Ca $(NO_3)_2.4H_2O$	1.22	1.22	1.22	1.22	1.22
NaFeEDTA	21.70	21.70	21.70	21.70	21.70
KI	4.50	4.50	4.50	4.50	4.50
$MnCl_2.4H_2O$	30.30	30.30	30.30	30.30	30.30
$ZnSO_4.7H_2O$	9.20	9.20	9.20	9.20	9.20
H_3BO_3	24.00	24.00	24.00	24.00	24.00
$CuSO_4.5H_2O$	0.50	0.50	0.50	0.50	0.50
$Na_2MoO_4.2H_2O$	0.01	0.01	0.01	0.01	0.01
KH_2PO_4	**35.00**	**10.00**	**20.00**	**30.00**	**40.00**
Glycine	40.00	40.00	40.00	40.00	40.00
Thiamine HCl	0.30	0.30	0.30	0.30	0.30
Pyridoxine HCl	0.50	0.50	0.50	0.50	0.50
Niacin	4.00	4.00	4.00	4.00	4.00
Myoinositol	277.00	277.00	277.00	277.00	277.00
Sucrose	10000	10000	10000	10000	10000

Supplementary table S3: Sand physical properties and water holding capacity

Sand Grade	Bulk density (gcm^{-3})	Solid particle density (gcm^{-3})	% Pore Space	Soil water content (volume of water cm^3/cm^3 dry sand)	Maximum Saturation capacity (SC), g/100 g	Field Capacity (FC), g/100 g	Wilting percentage (WP), g/100 g	Available water holding capacity (AWC) of sand at 0 - 18cm depth (cm)
Grade A	1.56	3.01	48.17	0.333	28.35	09.12	1.51	2.13
Grade B	1.39	2.53	45.06	0.357	29.43	15.46	6.23	2.31
Grade C	1.31	2.36	44.49	0.361	30.16	17.68	7.65	2.37
Grade D	1.26	2.29	44.98	0.400	33.79	22.97	8.73	3.23

Note: Available water Holding capacity (AWC), cm = Sand depth X Sand bulk density X ((FC/100)- (WP/100) cm

Supplementary table S4: Composition of Hoagland's nutrients solution

	Final Concentration per liter media	
Nutrient	Half strength	Full strength
$Ca(NO_3)_2.4H_2O$	2.5 mM	5 mM
KNO_3	2.5 mM	5 mM
$MgSO_4.7H_2O$	1.0 mM	2 mM
KH_2PO_4	**20 µM**	**40 µM**
NaFeEDTA	50 µM	0.1 mM
$Na_2MoO_4 \cdot 2H_2O$	0.2 µM	0.2 µM
H_3BO_3	10 µM	10 µM
$NiSO_4.6H_2O$	0.2 µM	0.2 µM
$ZnSO_4 \cdot 7H_2O$	1.0 µM	1.0 µM
$MnCl_2.4H_2O$	2.0 µM	2.0 µM
$CuSO_4 \cdot 5H_2O$	0.5 µM	0.5 µM
$CoCl_2.6H_2O$	0.2 µM	0.2 µM
MES buffer	0.5 mM	0.5 mM

Supplementary table S5: Hoagland's nutrient application

Sand Grade	Treatment	Application Duration (Range of Days)	Demineralized Distill Water Application Time 9.00 AM (ml/Kg sand)	Hoagland Solution Application Time 4.00 PM (ml/ Kg Sand)			
				Full strength	Full strength (- P)	Half strength	Half strength (- P)
A	T1	01 to 50 days	40	20	-	-	-
		51 to 90 days	40	20	-	-	-
	T2	01 to 50 days	40	20	-	-	-
		51 to 90 days	40	**-P**	20	-	-
	T3	01 to 50 days	40	-	-	20	-
		51 to 90 days	40	-	-	20	-
	T4	01 to 50 days	40	-	-	20	-
		51 to 90 days	40	-	-	**-P**	20
B	T1	01 to 50 days	40	20	-	-	-
		51 to 90 days	40	20	-	-	-
	T2	01 to 50 days	40	20	-	-	-
		51 to 90 days	40	**-P**	20	-	-
	T3	01 to 50 days	40	-	-	20	-
		51 to 90 days	40	-	-	20	-
	T4	01 to 50 days	40	-	-	20	-
		51 to 90 days	40	-	-	**-P**	20
C	T1	01 to 50 days	40	20	-	-	-
		51 to 90 days	40	20	-	-	-
	T2	01 to 50 days	40	20	-	-	-
		51 to 90 days	40	**-P**	20	-	-
	T3	01 to 50 days	40	-	-	20	-
		51 to 90 days	40	-	-	20	-
	T4	01 to 50 days	40	-	-	20	-
		51 to 90 days	40	-	-	**-P**	20
D	T1	01 to 50 days	40	20	-	-	-
		51 to 90 days	40	20	-	-	-
	T2	01 to 50 days	40	20	-	-	-
		51 to 90 days	40	**-P**	20	-	-
	T3	01 to 50 days	40	-	-	20	-
		51 to 90 days	40	-	-	20	-
	T4	01 to 50 days	40	-	-	20	-
		51 to 90 days	40	-	-	**-P**	20

Note: Treatment

T1= Full-strength Hoagland's solution with 40 µM P;

T2= full strength Hoagland with P omission (-P);

T3 = Half-strength Hoagland's solution with 20 µM P;

T4 = half-strength Hoagland with P omission (-P)

Supplementary table S6: Average spore size of *R. irregularis* grown in M-Medium supplemented with different level of P. Data are means ± SE (n = 10).

Treatment	Minimal (M) media	phosphorus (P) concentration	Average Spore size (µm)
T1	Modified M-media (40 µM KH2PO4)	40 µM KH2PO4	61±13
T2	M-media (control)	35 µM KH2PO4	58±13
T3	Modified M-media (30 µM KH2PO4)	30 µM KH2PO4	57±11
T4	Modified M-media (20 µM KH2PO4)	20 µM KH2PO4	52±11
T5	Modified M-media (10 µM KH2PO4)	10 µM KH2PO4	46±10

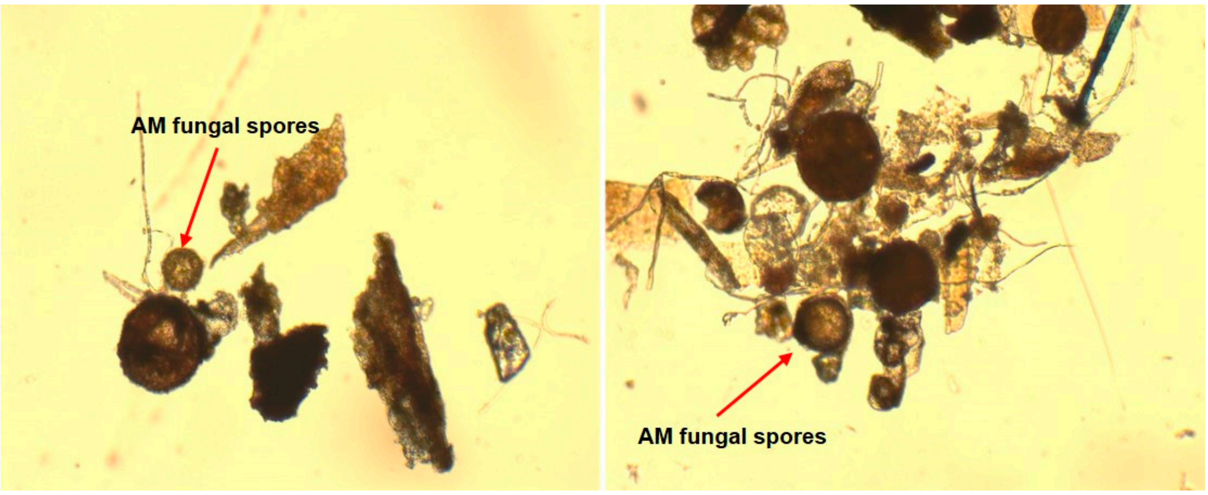

Supplementary figure S1: Arbuscular mycorrhizal (AM) fungal spores isolated from soil.

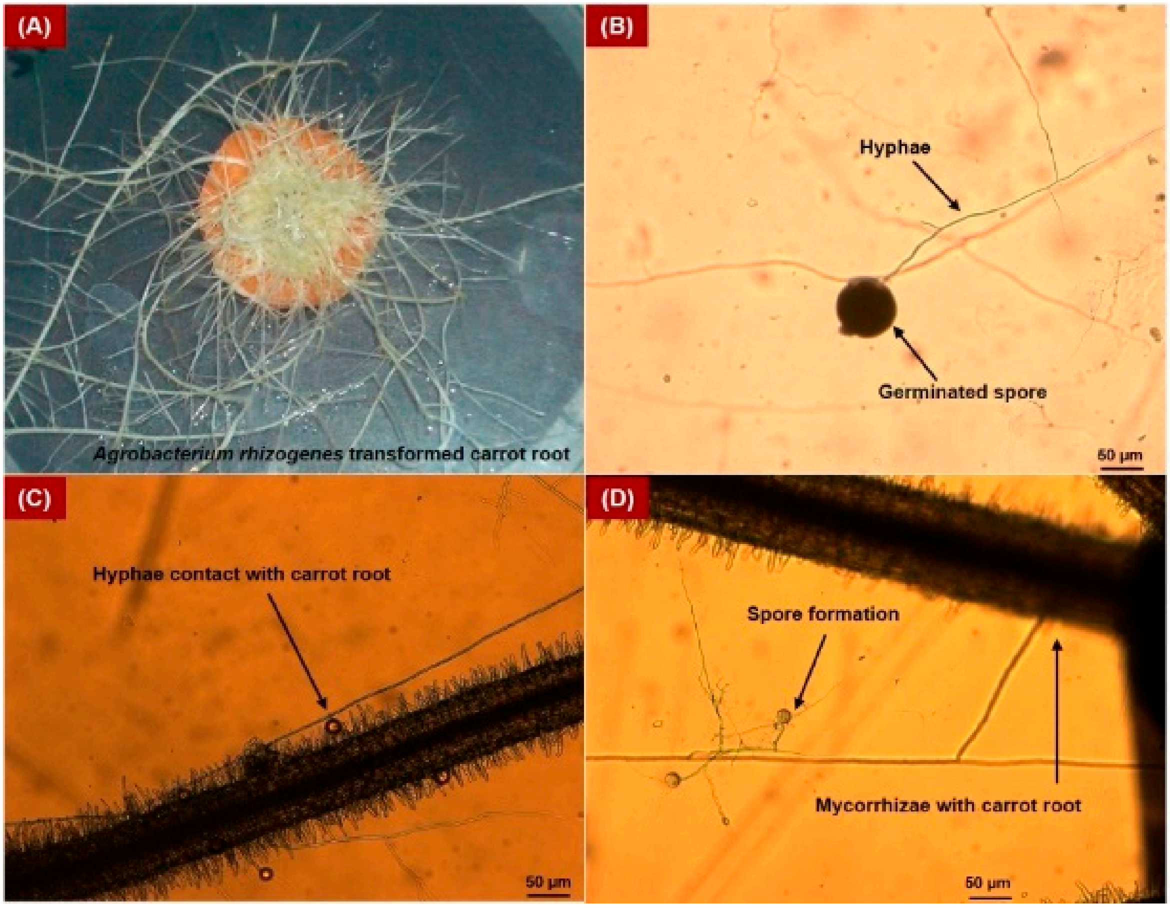

Supplementary figure S2. Establishment of monoxenic culture of AM fungi. (A) Induction of *A. rhizogenes* (ICMP8640) transformed carrot hairy root; (B) Germination of sterilized spore in M medium; (C) Mycorrhizal (germinated spore) in contact with carrot hairy root; (D) Mycorrhization of transformed carrot root

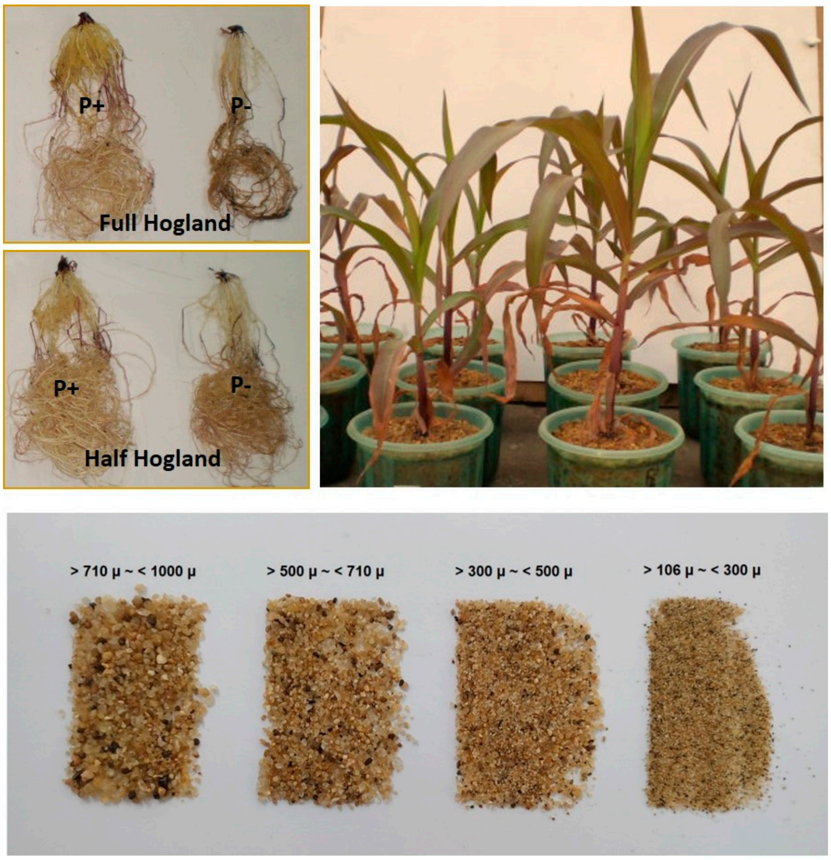

Supplementary figure S3: Illustration of pot experiment of maize plant inoculated with *R. irregularis* in different sand grade of sand.

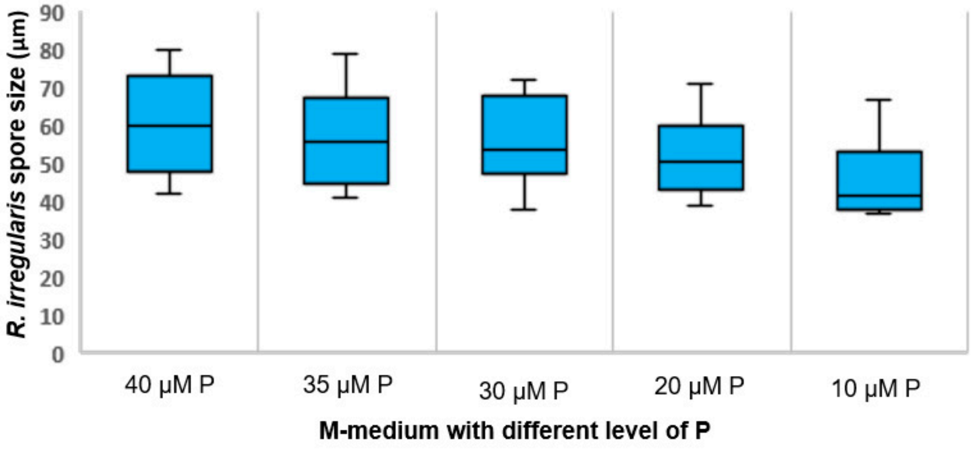

Supplementary figure S4: Box-plot described the spore size of *R. irregularis* grown in M-Medium supplemented with different level of P. The black line inside each box represents the median value (n= 10)
